# Omics-Based Approaches in Research on Textile Dye Microbial Decolorization

**DOI:** 10.3390/molecules29122771

**Published:** 2024-06-11

**Authors:** Anna Jasińska, Aleksandra Walaszczyk, Katarzyna Paraszkiewicz

**Affiliations:** 1 Department of Industrial Microbiology and Biotechnology, Faculty of Biology and Environmental Protection, University of Lodz, 90-237 Lodz, Poland; katarzyna.paraszkiewicz@biol.uni.lodz.pl; 2 Department of Industrial Microbiology and Biotechnology, Faculty of Biology and Environmental Protection, Doctoral School of Exact and Natural Sciences, University of Lodz, 90-237 Lodz, Poland; aleksandra.walaszczyk@edu.uni.lodz.pl

**Keywords:** textile industry, dyes, biodegradation, microorganisms, omics

## Abstract

The development of the textile industry has negative effects on the natural environment. Cotton cultivation, dyeing fabrics, washing, and finishing require a lot of water and energy and use many chemicals. One of the most dangerous pollutants generated by the textile industry is dyes. Most of them are characterized by a complex chemical structure and an unfavorable impact on the environment. Especially azo dyes, whose decomposition by bacteria may lead to the formation of carcinogenic aromatic amines and raise a lot of concern. Using the metabolic potential of microorganisms that biodegrade dyes seems to be a promising solution for their elimination from contaminated environments. The development of omics sciences such as genomics, transcriptomics, proteomics, and metabolomics has allowed for a comprehensive approach to the processes occurring in cells. Especially multi-omics, which combines data from different biomolecular levels, providing an integrative understanding of the whole biodegradation process. Thanks to this, it is possible to elucidate the molecular basis of the mechanisms of dye biodegradation and to develop effective methods of bioremediation of dye-contaminated environments.

## 1. Introduction

The textile industry is one of the largest and most diverse industrial sectors in the world, annually producing millions of tons of textiles to meet consumer demand worldwide. According to a report by InkWorld [1], the global textile market was valued at USD 961.5 billion in 2019, with an anticipated expansion at a compound annual growth rate of 4.3% by 2027. These numbers reflect the importance of the textile industry to the world economy. This massive production scale involves various sub-sectors starting from the production of fibers and yarns and ending with the distribution of ready-made clothes and other textile products (including carpets, sheets, towels, upholstery, textile bags, and products for special purposes, e.g., medical or military) [2].

However, textile production has a devastating impact on the environment [3]. It consumes substantial amounts of water and land to grow cotton and other natural fibers. Additionally, agricultural production requires fertilizers and pesticides, which may have a toxic effect on living organisms, including humans [4,5,6]. Often, these are complex chemical compounds that are difficult to biodegrade, and once introduced into the environment, they can accumulate, e.g., in the soil or animal tissues. Among the pesticides used in cotton production, the most common are insecticides such as organophosphates, pyrethroids, and neonicotinoids, e.g., endosulfan, aldicarb, imidacloprid, and glyphosate [7,8,9]. Pesticides are easily spread in the environment through the air and water, often over very long distances. Many of them remain in the soil and take a very long time to decompose. Due to their persistence in the soil, their toxicity, and high biological activity, they pose a threat to the environment. The most important negative features of pesticides include the phenomenon of pests becoming resistant to the preparations used [10]. Harmful chemicals are also used at subsequent stages of textile production posing a threat to humans when they enter the environment through waste and sewage. It is estimated that textile production is responsible for approximately 20% of global clean water pollution, due to the dyeing and finishing of products [11,12]. The attention of researchers dealing with pollution generated by the textile industry is mainly drawn to synthetic dyes. Dye molecules contain in their chemical structure groups of atoms that are responsible for the color and are called chromophores. Based on the chemical structure, 20–30 different dye groups can be identified, the most important of which are azo, anthraquinone, phthalocyanine, and triarylmethane dyes [13]. It is estimated that approximately 10–15% of dyes remain unbound from materials during dyeing and end up in sewage, which is not always fully treated in sewage treatment plants. Untreated synthetic dyes cause coloration of water bodies, which reduces light penetration through water and decreases photosynthetic activity [14]. Many dyes have carcinogenic, mutagenic, and teratogenic properties and demonstrate toxic impacts on plants, animals, and humans [15].

The negative impact on the environment is further increased in the case of fabrics manufactured using synthetic fibers such as polyester, acrylic, polyamide, or nylon, which make up about 60% of clothing and 70% of household textiles [16]. During use, especially washing, these materials release microscopic plastic particles called microplastics [17]. Microfibers are discharged in sewage to treatment plants, but due to their synthetic nature; they do not biodegrade and are discharged into water with treated sewage. They can also get into the soil, e.g., with sewage sludge, which is often used to fertilize fields and meadows [18,19].

To get rid of pollutants emitted by the textile industry, chemical, and physical methods are used, most of which are effective and easy to apply, but may not always be cost-effective and environmentally friendly [20,21,22]. Additionally, dyes representing various chemical groups are present in the sewage. Industrial wastewater contains other chemicals in addition to dyes, so many of these treatment methods may not be effective and may need to be combined with another treatment method to achieve maximum color removal. However, biological methods that use fungi, bacteria, or enzymes isolated from them to degrade chemical compounds generated from the textile industry are extremely promising [23]. Biological methods of dye elimination are considered the best due to their ability to eliminate various chemical classes of dyes, generate less volume of sludge, and are a cost-effective and safer approach to the disposal of textile wastewater.

However, in order to be able to use biological methods to remove dyes from contaminated environments, it is necessary to thoroughly understand the nature of the processes occurring in microbial cells in response to contact with the dye/dyes. For this reason, in the present work, after outlining the threats to the environment and humans posed by the textile industry, we focus on the use of biological methods to remove one of the largest groups of pollutants in the textile industry—dyes. Special emphasis is placed on discussing the latest research using omics sciences such as genomics, transcriptomics, proteomics, and metabolomics, which provides thousands of pieces of information about processes occurring in the cell and in the extracellular environment. Extensive results obtained through the application of omics techniques allow effective methods for the biodegradation of textile pollutants and their practical implementation to be developed.

## 2. Global Textile Industry and Its Environmental Footprints

**Figure 1:**
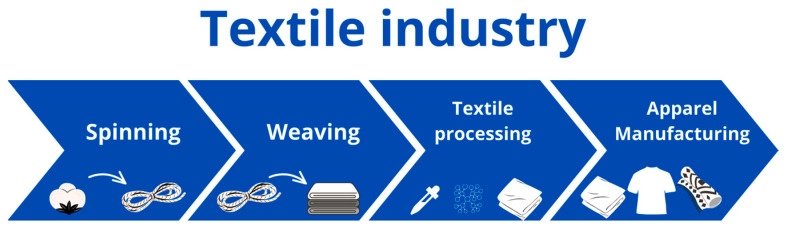
The flow chart of the textile manufacturing process.

The textile industry is one of the most important and oldest industries in the world. The main sub-sectors of the textile industry, representing key textile manufacturing processes, are spinning (the process of converting fibers into yarn), weaving (the weaving or knitting of yarn into fabrics), textile processing (the dyeing and printing of fabrics before processing them into the final product), and apparel manufacturing (transforming fabric into clothing or another textile product) [24] (Figure 1).

The fabrics applied in textile products can vary greatly and include natural (obtained entirely from products of vegetable origin, e.g., cotton, linen, hemp, and jute or animal origin, e.g., wool and silk), artificial (derived from materials of natural origin, but processed through chemical transformation, e.g., viscose, cellulose, acetate, rayon), and synthetic fibers (produced by chemical synthesis using petroleum by-products and materials of mineral origin, e.g., nylon, polyester, polyamide, acrylic) [25]. Virgin fossil-based synthetic fibers have dominated the fiber market since the mid-1990s when they overtook cotton volumes. The wide use of synthetic fibers is due to their beneficial properties such as high strength, chemical and wrinkle resistance, quick-drying, high elasticity, and moisture-absorbing properties.

Textile industry branches together play an important role in the global economy. The global textile market was valued at USD 1695.13 billion in 2023 and is anticipated to grow at a compound annual growth rate (CAGR) of 7.6% in revenue from 2023 to 2030. As much as 72.4% of revenues generated from textiles in 2022 came from the sale of fashion and clothing, followed by household, technical, and others [26]. According to the European Parliament report (environmental impact of the textile and clothing industry), about 5% of the household income of EU citizens is spent on clothing and footwear. For example, in 2015 EU citizens bought 6.4 million tons of new clothing (which gives 12.66 kg per person) [27]. Moreover, the total global consumer spending on clothing and footwear is estimated to increase by almost 20.5% between 2024 and 2029, and reach USD 2.9 trillion in 2029 [28]. The demand for textile industry products is still growing, driven by, among others, the fast fashion industry, which is the business model of the clothing industry consisting of the mass production of clothes, accessories, and footwear, and which is distinguished by very low prices and response to the most popular trends in fashion [29].

**Figure 2:**
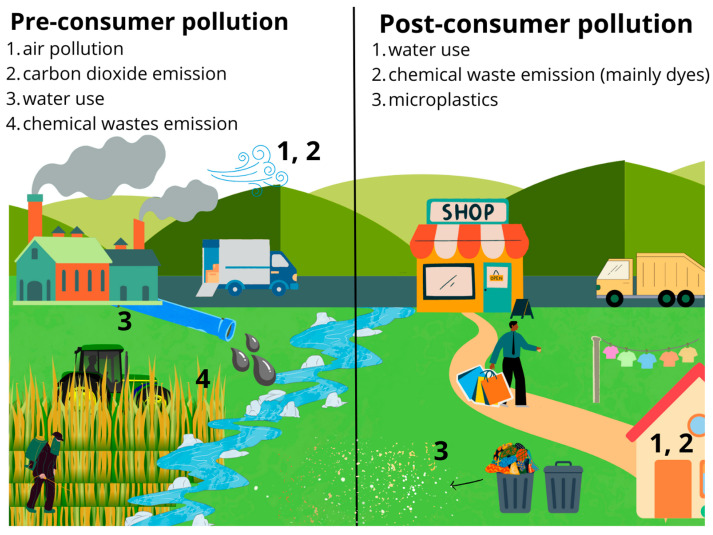
Pre- and post-consumer dangers associated with the production and use of textile industry goods.

Unfortunately, the textile industry is considered one of the most polluting industries (Figure 2). Each sub-sector of the textile industry has a negative impact on the natural environment [30]. Firstly, the cultivation of plants whose fibers are used to produce textiles requires huge areas of agricultural land. Moreover, these crops require intensive irrigation and pesticide consumption [31,32]. Spinning and weaving use solvents, surfactants, lubricants, bleaches, softeners, anti-foaming agents, and durable water repellents. Also, in the dyeing process, a huge amount of water is required compared to other processes (10–300 L/kg of product). At this stage of production, hazardous chemical substances are additionally used, such as fragrances, dyes, brominated flame retardants, stain repellents, antibacterial agents, and phthalates [33,34]. It is known that the textile industry takes advantage of over 3500 substances including 750 classified as hazardous for human health and 440 as hazardous for the environment. These substances are discharged in sewage to treatment plants, but due to imperfections in the treatment processes, they then enter the natural environment, causing 20% of global water pollution and adversely affecting the organisms living there [35]. For example, during dyeing processes, as much as 10–15% of the dyes are not bound by the fibers of dyed materials, which results in huge amounts of colored wastewater being released each day into the sewage system or directly into the environment [36]. According to analyses by the Intergovernmental Panel on Climate Change, the textile industry causes 10% of global greenhouse gas emissions. The European Environment Agency reports that textile production emits 15–35 tons of CO_2_ equivalent for every ton of fabric produced.

However, the responsibility for the destructive impact on the environment lies not only with companies associated with the textile industry. Users of fabrics, clothes, and home textile products also emit pollutants during their use. The dramatic development of fast fashion and the ever-increasing volume of textile consumption have contributed to shortening the service life of clothes and resulted in an increase in the amount of textile waste generated. The average resident of the European Union throws away nearly 11 kg of clothing per year [37]. Currently, only 1% of textile waste is recycled into new clothes. Most textile waste is disposed of by incineration or deposited in landfills (87%). Huge amounts of clothes and other textiles from the EU and the USA go to Africa or South America, for example, in the Atacama Desert of Chile [38]. Hazardous waste is also released when clothes are used. During washing dyes, chemicals, and microplastics get into household waste water. About half a million tons of plastic microfibers are released into the ocean annually from washing textiles made of synthetic fibers such as polyesters or polyamides. It is estimated that approximately 8% of microplastics polluting the oceans comes from textile use in Europe. This amounts to approximately 13,000 tons of textile microfibers ending up in surface waters (which is 25 g per person). The largest amounts of microfibers are released during the first few washes. Combining this fact with the intensive development of fast fashion, which often uses polyester and lasts for a shorter time, the phenomenon is very disturbing [17,39,40]. Figure 2 summarizes both pre- and post-consumer dangers associated with the production and use of textiles.

In the following chapters, we will focus on dyes as pollutants whose elimination may potentially be possible using methods that take advantage of the metabolic potential of microorganisms.

## 3. Dyes—Characteristics, Applications, and Danger

Dyes are compounds that give color when bound to a material, absorbing light in the visible spectrum due to chromophores, conjugated systems, and electron resonance. They contain auxochromes influencing their color and solubility. There are more than 100,000 commercially available dyes with over 7 × 10^5^ tons produced per year. According to Synthetic Dyes Global Market Report 2022, the global synthetic dye market was worth USD 6.3 billion in 2022, with China and India as the key producers [41].

Among dozens of chemical classes of dyes that differ in their chemical structure, the most popular are azo, anthraquinone, phthalocyanine, and triarylmethane dyes [42]. Azo dyes contain one or more (usually four) azo groups (–N = N–) linked to phenyl and naphthyl radicals usually replaced with some combinations of functional groups including amino, chlorine, hydroxyl, methyl, nitro, and others. Azo dyes are widely used not only in the textile industry but also in printing and paper manufacturing. Color intensity and variety, good dyeing properties, and stability under light, heat, and various chemicals are the main properties that ensure the durability of colors in different conditions [43]. Anthraquinone dyes commonly used for the coloration of cellulose fibers and cotton as well as synthetic fibers of hydrophobic nature (such as polyester, acetate, and nylon) have similar properties [44].

Due to imperfections in the technological operations of coloring processes, huge quantities of dyes are released and pollute the environment. The amount of discharged dyes is variable and, depending on the type of the dye, it can reach from 2 to 50%. Textile wastewater containing dyes, when released into the environment, causes visible water discoloration, limiting its usability for humans and animals. Even at low concentrations, dyes hinder sunlight penetration, affecting aquatic plant photosynthesis and disrupting gas solubility, leading to fish and microorganism deaths and ecosystem dysfunction [45]. Moreover, in developing countries using textile wastewater for crop irrigation, dyes can contaminate soil, altering its biological properties and enzyme activity. Dyes degrade soil quality, reduce agricultural productivity, and threaten food safety. For instance, Topac et al. [46] demonstrated a significant decline in soil quality due to sulfonated azo dye Reactive Black 5, leading to decreased bacterial activity and nitrogen-use efficiency, further impacting terrestrial ecosystems. What is more, many dyes are highly stable in natural environments, with half-lives varying from a few days to months or even years [23].

Dyes can cause various health issues, including indigestion, anemia, asthma, and urticaria. They might also affect neurotransmitters and lead to microscopic brain damage, potentially resulting in neurological disorders [23]. Additionally, dyes have been linked to pathological lesions in organs like the kidney, spleen, and liver, as well as cancer, abnormalities in offspring and growth retardation. Many dyes are considered priority pollutants by the United States Environmental Protection Agency (US EPA) due to their toxic, mutagenic, and carcinogenic properties [15]. Azo dyes widely used in the textile industry are of particular concern due to their acute and chronic effects on organisms and their potential to be converted by intestinal and skin bacteria into carcinogenic aromatic amines. Bacteria species such as *Staphylococcus*, *Corynebacterium*, *Micrococcus*, *Dermacoccus,* and *Kocuria* have been reported to produce these carcinogenic amines from azo dyes [47].

Most toxic dyes are banned, like Sudan dyes used in spices and flavorings, classified by the International Agency for Research on Cancer (IARC) as carcinogenic. For example, Mohamed et al. [48] found that Sudan III adversely altered the hepatic enzymes, lipid, and oxidative stress biomarkers in the liver of rats when exposed to 125 or 250 mg/kg body weight. Another azo dye, Sunset Yellow, administered orally to rats for 40 days adversely affected brain tissue by causing oxidative damage [49]. But azo dyes are not the only compounds that can have a toxic impact on living organisms. A similar effect may be exerted by the products of their anaerobic decomposition—aromatic amines. The EU restricts azo dyes breaking down into 22 carcinogenic aromatic amines. REACH legislation formulated by the European Commission (Section 43 of Annex XVII of REACH, Azo dyes, and Azo colorants) prohibits azo dyes in consumer products (such as clothing, bedding, towels, nappies, textile or leather toys, purses/wallets, bags, yarn, and fabrics) if they produce one or more of the known carcinogenic aromatic amines in detectable concentrations, (defined as 30 ppm) [50]. Brüschweiler et al. [50,51] found that among the 896 azo dyes available in the textile dyes database, 426 (48%) can metabolize into EU-regulated aromatic amines, while 470 (52%) can produce other hazardous amines. Researchers warn about dyes, especially benzidine-derived ones, potentially disrupting the endocrine system. Bazin et al. [52] found some dyes with estrogenic or anti-estrogenic activity, suggesting they may function as endocrine disruptors.

## 4. Non-Biological Treatment Methods for Textile Dye Elimination

The places where textile dye contamination is most noticeable and undesirable are water bodies. According to the United Nations World Water Development Report, nearly 60% of the world’s population will have a problem with a shortage of clean water by 2050 [53]. Due to the lack of technological solutions and the large scale of production of textiles that are then exported to external markets, the situation is particularly disturbing, especially in middle- and low-income countries. Therefore, intensive research is underway to develop effective methods of eliminating pollutants such as textile dyes.

Textile dyes can be removed from sewage and polluted water bodies by physical methods based on the mass transfer mechanism using mainly the processes of adsorption, ion exchange, and membrane filtration. These technologies are characterized by simplicity of construction and operation, low costs, and quite high universality in relation to the pollutants eliminated. Their effectiveness is relatively high and ranges from 85% to 99%. However, the use of these methods may generate toxic by-products and sediments and may be difficult under certain conditions (e.g., high temperatures and the presence of heavy metals). On the other hand, chemical methods such as coagulation, flocculation, and oxidation are usually expensive, characterized by high demand for electricity, large amounts of chemicals used, and advanced equipment. Moreover, they may also produce toxic metabolites and by-products. A thorough characterization of physicochemical methods applied to eliminate toxic pollutants from the textile industry has been recently presented by Holkar et al. [21], Azanaw et al. [22], and Roy et al. [23].

## 5. Microorganisms as a Tool for Textile Dye Elimination

Microorganisms and their enzymes (e.g., laccases, peroxidases, and azoreductases) offer an alternative for textile pollutants elimination where dyes can be directly degraded or transformed into less harmful compounds. More than 80,000 papers on dye biodegradation have been published in the last twenty years, focusing on the screening of microorganisms active in dye elimination, biodegradation kinetics and pathways, mechanisms, formed intermediates, and final products. There are many reasons for such great interest in these biological methods. This approach offers low-cost solutions that are easy to implement and generate minimal amounts of waste. Microorganisms can thrive in various conditions, making this method versatile and scalable. However, for practical implementation of solutions based on the metabolic activity of microorganisms, it is necessary to meet various challenges, including understanding molecular mechanisms controlling the biodegradation process, identifying the most effective enzymes, and ensuring optimal environmental conditions for microorganisms (especially for real wastewater where dyes coexist with other pollutants that may inhibit the activity of enzymes, limiting the effectiveness of the process) [21].

There are two groups of microorganisms that are particularly useful in the microbial treatment methods. The first group consists of indigenous strains living in contaminated soil or water and able to survive in it thanks to the development of adaptation mechanisms that neutralize the pollutants. For example, Jasińska et al. [54] isolated tolerant fungal strains from soil around the textile dyeing factory and tested their potential to remove different industrial dyes. Strains of *Penicillium pinophilum* and *Myrothecium roridum* almost completely eliminated Malachite green within 48 h. Similarly, Modi et al. [55] isolated and assessed the ability of bacteria from dye house effluent to decolorize dyes. Among seven isolates, *Bacillus cereus* was found to be the best decolorizer for tested sulfonated azo dyes among all isolates. Another popular approach is the purposeful acclimatization of microorganisms, mainly bacteria, to unfavorable conditions, i.e., the presence of toxic dyes. During this process, the microbial population is constantly exposed to the dye, which results in a more rapid transformation (biodegradation) of the compound than initially observed. For example, Karishnan et al. [56] investigated the biodegradation of a mixture of three azo dyes using a mixed bacterial culture isolated from the sludge derived from an effluent treatment plant and acclimatized prior to the experiment to obtain a culture with an effective degradation ability. Obviously, not every strain isolated from a dye-contaminated environment will be able to decolorize dyes, just as strains not previously exposed to such contaminants may turn out to be excellent decolorizers. In the research conducted by Seyedi et al. [57], from 50 strains of bacteria isolated from the water and soil samples collected from areas around the textile plant, only three bacterial strains exhibited high decolorization abilities for Reactive Black 5 and Reactive Red 152 dyes. Srinivasan and Sadasivam [58] compared the degradation of three azo dyes by non-adapted (acquired from a strain collection) and adapted (isolated from textile effluent polluted soil) strains of *Aeromonas hydrophila* and found that both adapted and non-adapted bacteria functioned as azo dye degraders.

The second group of microorganisms useful in dye degradation comprises fungi-producing ligninolytic enzymes that degrade lignin and related aromatic compounds. However, these enzymes are also capable of degrading a diverse range of persistent organic pollutants including dyes. Ligninolytic enzymes include extracellular non-specific and non-stereoselective lignin peroxidases (LiP), manganese peroxidases (MnP), and laccases. Their participation in dye degradation processes has been summarized by Herath et al. [59]. The use of crude or isolated and purified enzymes is also extremely promising. Efficient decolorization of RBBR dye by *Arthrographis kalrae* laccase combined with a decrease in phyto- and cytotoxicity has been recently presented by Yadav et al. [60]. Similarly, crude thermostable laccase from *Pycnoporus* sp. SYBC-L3 decolorized several disperse dyes with efficiency dependent on the dye type, concentration, enzyme loading, and mediator presence, ranging from 51 to 96% [61]. Crude laccase from the fungal strain *Peroneutypa scoparia* was successfully applied for the decolorization of eight leather dyes [62]. Ligninolytic fungi also synthesize versatile peroxidase (VP) and dye-decolorizing peroxidase (DyP). VP is an enzyme with broad substrate specificity, combining the catalytic properties of manganese peroxidase and lignin peroxidase. It oxidizes phenolic and non-phenolic lignin compounds, aromatic compounds, and different industrial dyes. DyP is highly effective at degrading synthetic dyes, particularly those that are recalcitrant to breakdown by other enzymes [63]. In the case of azo dyes, fungi- and bacteria-producing azoreductases are often used. These are flavo-enzymes involved in the reduction of azo bonds [64].

Modern analytical techniques working for the needs of the so-called omics sciences offer insights into microbial degradation mechanisms, aiding in developing efficient bioremediation strategies. For example, genomics and transcriptomics provide information about specific genes encoding enzymes able to degrade the dyes, metabolomics informs about the metabolites formed during the microbial reactions, and proteomics can help characterize enzymes engaged in these processes. Finally, integrative “omics”-based approaches open new opportunities to investigate the genome, transcriptome, proteome, and metabolome of microorganisms and decipher the molecular mechanisms of biodegradation. In the next part of this article, the traditional approach to research on the degradation of dyes by microorganisms will be analyzed and compared with a holistic omics-based approach to the process of eliminating pollutants.

### 5.1. Conventional Workflow

**Figure 3:**
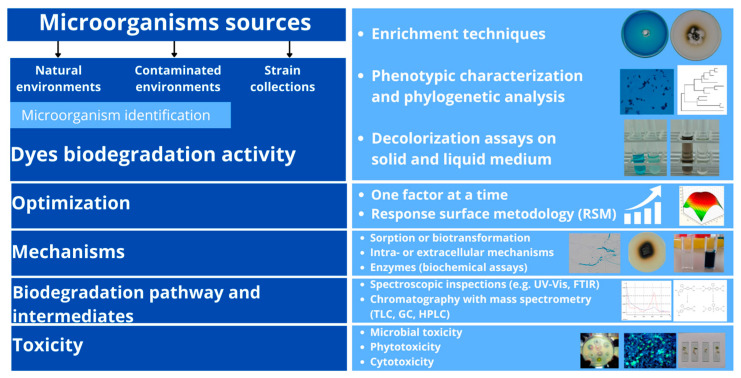
A conventional workflow of research for the development of a new, microbial-based solution for dye biodegradation.

Decolorization of dyes by microorganisms may take place in two ways: adsorption on the living or dead microbial biomass or intra-/extracellular biodegradation of dyes by the living cells. These aspects have been widely discussed by us previously [65]. Most works on the microbiological elimination of dyes from the beginning of the 21st century have been dominated by the traditional approach, which involves isolation and identification of microorganisms with potential biodegradation activity, optimization of biodegradation conditions, preliminary determination of the biodegradation mechanism, establishment of the dye biodegradation/biotransformation pathway, identification of emerging intermediates, and toxicity analysis (Figure 3).

Generally, screening of microorganisms with a potential dye biodegradation ability is performed using traditional isolation techniques. A solid support with the addition of a dye is often used for this purpose. The microorganisms around the colonies of which the zones of dye discoloration are observed are then isolated and used in further analyses. In addition, enrichment techniques using the medium containing a dye as a carbon source can be applied [66,67]. After isolation, the bacteria and fungi are identified mainly based on the phenotypic characteristics and phylogenetic analysis.

The biodegradation efficiency of dyes by microorganisms depends on many factors. These are the type and concentration of nutrients, pH, temperature, aeration, dye concentration, and presence of additional substances. To obtain the highest possible biodegradation efficiency in a relatively short time, process optimization is essential. In the classical “one factor at a time” approach, the process is optimized by changing one factor at a time while keeping the others at a constant level. However, this approach is extremely time-consuming, and expensive, and neglects the interaction between factors, which can lead to the wrong conclusion. A solution could be using experimental designs, for example, response surface methodology (RSM), whose advantage is the possibility of checking the simultaneous influence of many factors in the biotechnological process. RSM has been successfully applied for the optimization of dye biodegradation by microorganisms and their enzymes as well as in physicochemical processes [68,69].

To identify the mechanisms responsible for decolorization, the cultures are autoclaved to check whether the process is based mainly on sorption, or whether it requires living cells that actively metabolize the compound. Additionally, the activity of intra- and extracellular enzymes is examined. The contribution of enzymes such as azoreductases, laccases, or peroxidases is usually assessed using simple biochemical analyses. If their activity during the decolorization process is increased, it is concluded that they participate in the elimination of the dye [54,70,71]. Enzyme inhibitors and genetic modifications are also used, which inactivate gene-encoding proteins and enzyme systems (e.g., cytochromes) suspected of being involved in decolorization [72].

To assess the efficiency of the degradation process, it is necessary to monitor the intermediates and the final degradation products. The most frequently used techniques are spectroscopic inspection and separation of dyes and their intermediates by chromatography followed by identification with the use of mass spectrometry (MS). The basic spectroscopic technique is UV–vis (UV-vis) spectroscopy. In the case of decolorization, the sharp peak (at λ max) disappears and if a derivative is formed, an increase in absorbance can be observed in another UV region. Fourier Transform Infrared (FTIR) spectroscopy is also commonly applied. The FTIR spectrum makes it possible to determine both the type and strength of interactions occurring within azo dyes containing various functional groups, which shows structural changes in the dye molecule and the metabolites of dye degradation. Among the separation techniques, thin-layer chromatography (TLC) has been widely used due to its simplicity, resolving power, and speed. However, with the development of high-performance liquid chromatography (HPLC) and gas chromatography (GC), TLC has been superseded. Techniques for the analysis of dyes and their derivatives formed during decolorization with the participation of microorganisms have been widely discussed by Saratele et al. [73], Danouche et al. [74], and Kishor et al. [75].

Dye decolorization is not always synonymous with detoxification. Indeed, the disappearance of the color could not be directly related to toxicity reduction as the transformation products could be more toxic than the parent compounds. The best examples are azo dyes converted to aromatic amines, which are often more toxic than the parent dye. Rawat et al. [76] described almost complete decolorization of presumably non-toxic azo dye—Acid orange 7 (AO7)—to more harmful aromatic amines such as aniline, 1-amino-2-naphthol, naphthalene, and phenyldiazene. In contrast to AO7, these intermediates caused molecular, cellular, and organism-level toxicity. Similarly, the presence of highly toxic metabolites in the reaction medium was detected at the end of the treatment of the binary solution of Acid Blue 161 and Procion Red MX-5B by *Aspergillus tereus* [77]. Therefore, to fully characterize the ongoing process and its effects, it is necessary to assess the reduction in dye toxicity after decolorization. For this purpose, direct toxicity tests are used, involving indicator organisms, e.g., bacteria, microalgae, invertebrates, plants, and fish. Depending on the indicator organism used, toxicity is assessed, manifesting itself in growth inhibition, reduction in chlorophyll synthesis, increase in the level of oxidative stress, and growth and reproduction disorders. In addition, dyes and metabolites resulting from their decolorization can be subjected to genotoxicity tests on various cell lines. Methods of assessing toxicity have been discussed in detail by Ceretta et al. [78] and Danouche et al. [74].

### 5.2. Omics-Based Approaches in the Dye Biodegradation Research

The implementation of biological methods for the degradation of toxic textile dyes may be limited due to insufficient knowledge of the comprehensive mechanisms controlling this process. The development of the “omics” sciences has enabled integrated, high-throughput approaches that provide a wealth of information about microbes and their metabolism in a relatively short time. Thanks to this, it is possible to understand biochemical systems and their dynamic evolution, and to design methods favoring the effective degradation of xenobiotics.

**Figure 4:**
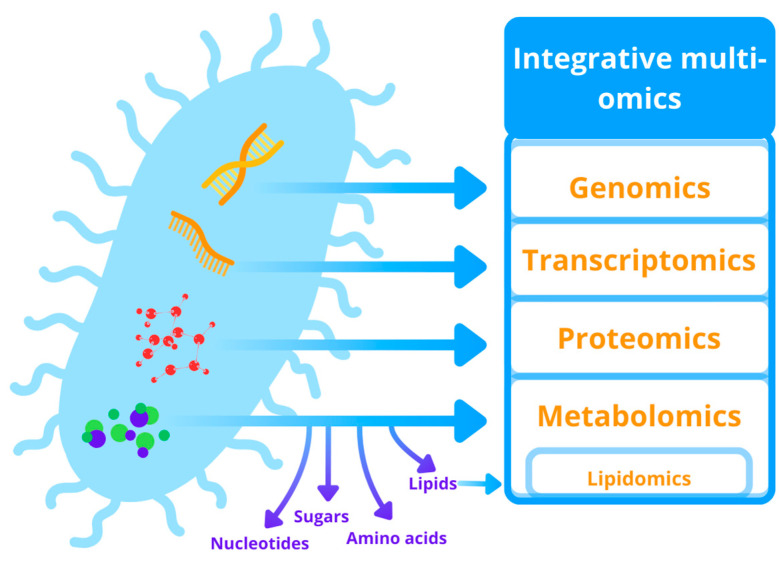
Omics techniques applied in research on microbial decolorization of dye pollutants.

Omics techniques include mainly genomics, transcriptomics, proteomics, lipidomics, and metabolomics (Figure 4). By using these techniques, it is possible to achieve a complete understanding of the microbial processes resulting in more effective bioremediation at the field scale [79]. There are two basic strategies for analyzing biological systems in omics research. The first one is a comprehensive, undirected analysis of samples, which does not require prior knowledge of the metabolic pathways, genes, or proteins of the examined microorganism. This strategy is called a shotgun approach and analyzes a wide range of molecules present in a sample, generating a huge amount of information that must be compared and correlated between different samples. The second strategy, called a targeted approach, is focused on research to identify specific compounds based on prior knowledge. Metaomics combines multiple genomics studies for horizontal meta-analysis and data integration. However, it is possible that information generated by a single-omics study is not always sufficient to fully understand a complex process such as microbial bioremediation; therefore, integrative multi-omics approaches are often required [80].

#### 5.2.1. Genomics and Transcriptomics

Omics techniques can already be applied at the stage of isolation from the environment of microorganisms used in dye decolorization processes. Genomics and metagenomics, which cover all genetic phenomena and define and describe their relationships, fitting them into the overall metabolic processes occurring in the cell, have opened new doors for research on the biodegradation of pollutants, including dyes. In the past decade, complete genome sequences of several dozen microorganisms significant to decolorization have been provided. Most of them are bacterial strains of the genera *Enterococcus*, *Citrobacter*, *Pseudomonas*, *Shewanella*, *Deinococcus,* and *Dehalococcoides*. Bacteria and fungi with decolorization potential in which the genome has been sequenced recently are summarized in Table 1.

Genome sequencing is most often carried out on bacteria isolated from contaminated environments to discover the possibility of using them in dye biodegradation and to identify the possible mechanisms of this process. However, it should be remembered that microorganisms under the influence of various chemical pollutants, including textile dyes, can modify gene expression to activate metabolic pathways responsible for neutralizing pollutants dangerous to the cell. The use of transcriptomics, i.e., the examination of the entire set of transcripts (all active RNAs), allows monitoring of the response of a microorganism in the presence of actual contamination. The use of transcriptomics can also help to understand the defense mechanisms of microorganisms against toxic substances and the impact of pollutants on cell physiology and metabolism. Transcriptomics is also used to assess the effectiveness of pollutant biodegradation by microorganisms and to search for new enzymes that degrade toxic substances. The application of genomics and transcriptomics to the identification of different promoters, genes, and degradation pathways has resulted in the construction of strains more efficient in the removal of pollutants. For example, Ye et al. [103] identified the gene Lac591, encoding a novel multicopper oxidase with laccase activity through activity-based functional screening of a metagenomics library from mangrove soil. Then, the gene was used to construct an *E. coli* mutant (Lac3T93) with a remarkably improved enzymatic activity [104]. Similarly, Shi et al. [94] applied genomics analysis of the endophyte strain *Pantoea ananatis* Sd-1 and identified in the bacteria genome laccase-encoding sequences, which were used to obtain a recombinant enzyme with an improved ability for dye decolorization and degradation of lignin. Very often, transcriptomics and genomics are used simultaneously to obtain a more complete look at the metabolic pathways taking place in the cell and to more precisely identify the factors responsible for the degradation and detoxification of dyes.

Chen et al. [90] applied genomics and transcriptomics techniques to elucidate the molecular mechanisms involved in azo dye decolorization by the thermophilic strain of *Anoxybacillus* sp. isolated from the soil near a textile dye manufacturing factory. The RNA-Seq and qRT-PCR technologies showed NAD(P)H-flavin reductase, 2Fe-2S ferredoxin, and NAD(P)-dependent ethanol dehydrogenase as the most likely factors involved in Direct Black G degradation.

Sun et al. [105] performed transcriptomics analysis during the degradation of Direct Red 5B azo dye by *Irpex lacteus* CD2 with lignin supplementation. They found a full spectrum of peroxidases and oxidases, radical-producing enzymes, and genes up-regulated under DR5B and lignin treatments. The transcriptomics analysis verified by selected protein over-expression assays indicated that aromatic dye degradation requires synergistic enzymes and radical-mediated oxidative reactions.

What is more, metagenomics and transcriptomics enable studying genomes without the need to cultivate microorganisms. Considering that unculturable microorganisms can constitute up to 99% of environmental strains and possess unique and potentially very useful abilities, it would be a big loss not to learn about them. Recently, Zhang et al. [106] applied shotgun metagenomics sequencing in the research on the suitability of a microbial community isolated from activated sludge in Reactive Black 5 biodegradation. The analysis allowed the researchers to characterize the ecological roles of the members of the community, to investigate the abundance and metabolic pathways of genes, and to propose the correlation of dominant species and functional genes. Guo et al. [107] used high-throughput sequencing to characterize the structure of the bacterial community from textile wastewater sludge and identified Bacillus as the most dominant genus, which played a key role in the decolorization process. Interesting results were obtained by Zhuang et al. [108] in the studies on the distribution of azo dye-degrading bacteria and their functional genes in estuaries and coastal environments of the Rongjiang and Hanjiang Rivers in eastern Guangdong (China). The researchers performed high-throughput sequencing (16S rRNA) of sediment DNA and demonstrated the presence of laccase, azoreductase (azoR), and naphthalene-degrading genes in the collected samples. The abundance of functional genes was higher in summer compared to winter, which may suggest that discharges from inland rivers influence the occurrence and abundance of azo dye-degrading genes in coastal environments. Decolorization of Reactive Brilliant Red X-3B wastewater with high salinity and metal ions presence by the microbial community acclimated from the surface soil of a pharmaceutical factory was investigated by Tan et al. [109]. The microbial community was found to efficiently decolorize even a high concentration of the dye (1100 mgL^−1^) in the presence of salt and some metal ions such as Mg^2+^, Ca^2+^, and Pb^2+^. The 16S rDNA-based fingerprint technique was used to investigate microbial population dynamics. The microbial community shifted during the acclimatization process affected by salt or metal ions. Some strains similar to *Bacillus*, *Sedimentibacter*, *Pseudomonas*, *Clostridiales*, *Streptomyces,* and some uncultured clones acted for the dynamic succession, supposedly as potential decolorization bacteria.

In summary, both genomics and transcriptomics are indispensable and highly effective tools for studying microbial community composition and activity during the decolorization of dyes. These techniques provide valuable insights into the crucial genes, enzymes, and metabolic pathways responsible for the degradation of dyes.

#### 5.2.2. Proteomics

Advances in genome sequencing enabled the identification of several open reading frames (ORFs) and provided extensive knowledge about genes that can influence the decolorization efficiency of bacterial and fungal cells. However, it should be remembered that the classical approach “one gene–one function” and/or “one gene–one enzyme” relationships are no longer applied since each gene may encode more than one protein based on differential splicing and translation factors. Thus, the number of genes present in the genome is smaller than the number of proteins present in the cell. For example, bacterial genomes encode approximately 600–6000 genes, but only about 50–80% of them are expressed in specific life circumstances, depending on environmental factors that influence the cell. However, proteomics studies allow us to analyze global protein expression in microbial cells. This means that we can determine which proteins, in what quantity, and order are produced by cells under specific environmental conditions, including in the presence of toxic factors.

Sun et al. [105] applied a shotgun proteomics technique to understand at the protein level the degradation mechanism of Direct Red 5B by the white rot fungus *Irpex lacteus* cultivated in the presence of lignin. About two thousand proteins were identified. The most predominant were enzymatic proteins from the oxidoreduction functional category. Based on these findings, it was concluded that the synergy of the Fenton reaction and manganese peroxidase might play an important role in the DR5B dye degradation. Shotgun proteome analyses of *Leptosphaerulina* sp. in dye decolorizing cultures demonstrated the presence of proteins homologous to diverse types of oxidoreductases, peroxidases, flavo-oxidases, and copper-containing metalloproteins [110]. Proteomics analysis of the proteins extracted during the azo dye decolorization by *Shewanella seohaensis* isolated from an estuarine environment was conducted by de Souza et al. [111]. The liquid chromatography–mass spectrometry–Quadrupole Time-of-Flight (LCMS-QTOF) analysis revealed that as many as 29 and 17 proteins were up-regulated during, respectively, 7 and 24 h of growth. The major class of up-regulated proteins included cellular oxidoreductases and an alkyl hydroperoxide reductase.

Mathur et al. [112] identified 90 intracellular and 13 extracellular proteins differentially expressed during cultivation of the fungus *Lentinus squarrosulus* AF5 in dye (mix of three different azo dyes)-treated conditions. The results of the proteomics study combined with biochemical tests allowed the selection of extracellular peroxidases as the main factors responsible for the decolorization of dyes. Proteomics techniques enable the differentiation and identification of intra- and extracellular (included in the secretome) proteins. For example, proteomics analysis of the secretome combined with biochemical tests using enzyme-specific substrates allowed Jasińska et al. [113] to conclude that extracellular laccases are responsible for the decolorization of azo dyes by the fungus *Myrothecium roridum*. Membrane proteome profiles were obtained by two-dimensional gel electrophoresis (2-DE) and matrix-assisted laser desorption/ionization time-of-flight–time-of-flight–mass spectrometry (MALDI-TOF/TOF/MS) for *Shewanella decolorationis* S12 cells grown with different amounts of amaranth [114]. Three proteins (specific kinase, protease, and reductase) involved in azo respiration and energy conservation in strain S12 were found. Table 2 summarizes the microbial proteins identified via proteomics study and proposed as being involved in dye decolorization.

Proteomics research is often supported by systems biology, which is concerned with integrating homology modeling, molecular docking, and investigating protein interactions to reveal the structure and function of enzymes in cellular metabolism. For example, outer membrane proteins (Omp(s)) were identified as crucial in azo dye decolorization by *Acinetobacter guillouiae* Ax-9 and *Rahnella aquatilis* [120]. Molecular modeling via SWISS-MODEL, and a structural comparison with well-studied channel-forming proteins from *Pseudomonas aeruginosa* (3SY7) provided by the Protein Data Bank, revealed that Omp(s) act as porins, efficiently absorbing small molecules and, through them, play vital roles in the biodegradation of azo dyes. Sharma et al. [121] adopted molecular docking to predict the catalytic interaction of the diazo dye, Congo red, with four dye enzymes (laccase, lignin peroxidase, azoreductase, and aryl alcohol oxidase) produced by the fungus *Trametes flavida*. Molecular docking of different dyes was also performed using laccase from *Rigidoporus lignosus* as a reference protein [122] and homology models for laccase and azoreductase enzymes of *Aeromonas hydrophila* SK16 and *Lysinibacillus sphaericus* SK13 [123,124]. The in silico results agreed with the results obtained during further in vitro studies. A molecular dynamics simulation was also employed by Haghshenas et al. [125] to investigate the interaction between selected azo dyes and azoreductase from mesophilic *Bacillus* sp. B29. They proved that azoreductase AzrC showed a high affinity toward hydrophobic dyes such as Acid Red 88. Molecular docking was also applied to assess FMN-dependent NADH-azoreductase from the bacteria *E. coli* with 18 azo dyes [126]. The use of molecular modeling and docking allows for the screening of pollutant’s susceptibility to degradation by already characterized enzymes and enables the preliminary selection of dye–microorganism–enzyme systems employed further in textile dye decolorization and degradation studies.

Proteomics also makes it possible to analyze microbial community structures, assess the dynamics of protein composition, and identify produced proteins. This approach is called “community proteomics” or “metaproteomic’’ and has also been employed to explore microbial diversity in pollutant-contaminated environments and characterize the metabolic potential for in situ bioremediation. Based on metaproteomics and metagenomics studies, Zhang et al. [106] proposed a molecular mechanism of RB5 degradation by candidate genes and found functional proteins of the dominant species creating a microbial community from the activated sludge collected from a sewage treatment plant. Similarly, An et al. [117] applied quantitative metaproteomics to identify the relative functional proteins involved in azo dye Direct Black G degradation by thermophilic microflora isolated from the soil. Although community proteomics regarding the decolorization of dyes is still in its infancy, its importance for studying the communities of microorganisms inhabiting various, including polluted, environments is increasingly appreciated. Recently, results obtained using metaproteomics have been reviewed by Wang et al. [127], Chandran et al. [128], and Mishra et al. [129].

Proteomics tools will enable scientists to precisely identify proteins and the dynamics of their expression during the biodegradation of pollutants, including dyes. Thanks to this, it is possible to identify key enzymes responsible for breaking down specific pollutants and discovering the metabolic pathways used by microorganisms during degradation, including in situ bioremediation in polluted environments. Knowledge obtained with proteomics tools helps to optimize bioremediation strategies.

#### 5.2.3. Metabolomics

In addition to identifying genes, transcripts, and proteins occurring in microorganisms at a given time under specific environmental conditions, metabolomics research is extremely valuable for identifying the processes occurring in the cell in the presence of textile dyes. The use of metabolomics allows for an even more complete understanding of the processes occurring inside cells (intracellular) and outside them (extracellular) in response to the action of dyes found in the growth environment. Metabolomics deals with the identification and quantitative analysis of low-molecular-weight (less than 1000 Da) products produced by cells at a given time and environmental conditions (including in response to pollution) [130]. Their identification over time allows us to determine the path the contaminant travels from the moment of contact with the microbial cell. Analysis of the metabolome over time allows for the indirect identification of metabolic pathways that are activated in the cell in response to the pollutant, and thus the determination of the biochemical activity of the microorganism [79].

Metabolomics is an extremely difficult field of science because a metabolome is a set of huge numbers of low molecular weight molecules. Moreover, it is dynamic and changes very quickly in a short period of time. Another problem is that unlike in the case of RNA (transcriptomics) and proteins (proteomics), it is not possible to demonstrate the connections of metabolites with specific genes. Additionally, the complex nature of cellular metabolism, in which the same metabolite may participate in many different pathways, complicates the interpretation of metabolomics data. Moreover, genome, transcriptome, and proteome analyses are based on targeted analyses of biopolymers with a relatively simple, specific composition (genome and transcriptome—four different nucleotides; proteome—22 amino acids) [130]. In the metabolome, the diversity of structures and chemical properties is much greater and includes inorganic ions, carbohydrates, volatile alcohols and ketones, amino acids, organic acids, hydrophobic lipids, and other complex chemicals. This makes it almost impossible to simultaneously determine the complete metabolome. Metabolomics analyses are most often performed in a way that aims to identify specific metabolites or metabolic pathways based on prior knowledge. Chromatographic methods are most often used to separate metabolites, often coupled with mass spectrometry, which enables the identification of separated metabolites based on databases and libraries. Strategies of the metabolomics approach in biodegradation study have been discussed widely by Rodríguez et al. [131], Lin et al. [132], and Callaghan [133].

Sun et al. [134] employed a UPLC/Qtof-MS metabolomics approach to investigate the metabolism of Sudan III and Orange II by *Staphylococcus aureus* as well as their effects on the metabolome of the bacterium. Dyes were metabolized mainly intracellularly but only Sudan III strongly affected the metabolome of the *S. aureus* cells. Among the detected 101 metabolites (including co-factors, amino acids, phenyl-containing organic acids, nucleotides, short-chain fatty acids, and others), eight metabolites significantly increased and 17 significantly decreased by Sudan III treatment, while only one metabolite production was significantly increased and three were significantly decreased by Orange II treatment. The most noticeable changes were observed among energy pathway-related metabolites. For example, a decrease in the content of 2-oxoglutarate (2-OG) and glutamate, polysaccharides (including maltotetraose and maltotriose) was observed, while lactose and hydroxybutyric acid (derived from ketosis) increased to compensate for 2-OG depletion in the Sudan III treatment. As a result, it was concluded that Sudan III might be comparatively more toxic than Orange II. Similarly, Zheng et al. [135] applied the untargeted LC–MS metabolomics approach for monitoring the degradation process of refractory dye Reactive Black 5 by *Klebsiella* sp. KL-1 and its recolorization in the presence of additional carbon and nitrogen sources. Metabolomics analysis performed with metabolite mass spectra databases such as HMDB (https://www.hmdb.ca/) and METLIN (https://metlin.scripps.edu/) showed changes in the level of 33 metabolites, of which the most significant differences were observed at the level of fatty acid amides and phospholipids. Cao et al. [136] conducted a 1H NMR metabolome analysis of *Shewanella oneidensis* MR-1 under azo dye decolorization conditions, which revealed significant alteration in the concentrations of nine metabolites. These metabolites were closely associated with glycolysis, amino acid metabolism, and DNA replication. Also, An et al. [137], using non-targeted metabolome analysis, found that the metabolic pathways associated with the fatty acid and amino acid biosynthesis and metabolism, together with the TCA cycle, were most significantly enriched during the bioremediation of papermaking black liquor by the bacterial strain of *Serratia* sp. AXJ-M. Li et al. [138] used the metabolomics approach during their investigation of azo dye wastewater decolorization in a microbial electrochemical system (MES) using different electrodes (e.g., graphene and polyaniline). Analysis has been performed using the UPLC system coupled to a quadrupole time-of-flight mass spectrometer and searching in databases such as the HMDB and Kyoto Encyclopedia of Genes and Genomes (KEGG) (https://www.genome.jp/kegg/). They found that decolorization is more rapid when the microbial community present in the MES is dominated by *Methanobrevibacter arboriphilus,* whose metabolites, including arginine and proline metabolism, purine metabolism, arginine biosynthesis, and riboflavin metabolism, are up-regulated when a graphene electrode is used. It was concluded that such conditions provided more electron shuttles and redox mediators, which facilitated the extracellular electron transfer and increased decolorization.

Further progress can be expected in research on dye biodegradation using a metabolomics approach. This will be related primarily to the development of analytical technologies, including increasing their resolution and sensitivity, using metabolic imaging techniques, integrating omics data obtained with various techniques, using artificial intelligence and machine learning, and developing microfluidic techniques. The abovementioned directions of development will allow for faster and more precise examination of the biodegradation of dyes at the level of single cells of microorganisms or microenvironments and, as a result, for a better understanding of the heterogeneity and dynamics of biodegradation processes.

#### 5.2.4. Integrative Omics

Advanced omics techniques offer a wide range of analyses and allow for overcoming problems and challenges that cannot be met by conventional biochemical and microbiological methods. Additionally, most of the contaminants are multi-component matrices; the breakdown of their individual components requires different metabolic pathways, sometimes occurring in different microorganisms that make up the population. Thanks to the use of genomics, transcriptomics, proteomics, and metabolomics, it is possible to obtain a comprehensive insight into the capabilities, molecular mechanisms, and interactions of cells and consortiums during the biodegradation of pollutants. However, data from individual omics levels cannot fully explain how these various multilayered biological processes interact with each other and how they lead to the occurrence of metabolic changes in cells that result in an increased capacity to biodegrade pollutants. For example, the use of genomics as a single-omics method does not provide information on the gene expression levels, protein functions, or metabolic activities. Similarly, transcriptomics used separately, without integration with other omics methods, does not reveal information about protein abundance, modifications, or metabolic states. Metabolomics as a single-omics method does not reveal the underlying genetic or protein-related causes of observed metabolic changes. To avoid false inferences resulting from missing or incomplete information, scientists use various omics techniques in parallel (so-called multi-omics/integrative omics approach) to obtain a more complete insight into the biodegradation processes taking place in the cell.

The application of a multi-omics approach integrates different levels of data generated by genomics, transcriptomics, proteomics, and metabolomics, and reinforces complementary evidence from multiple levels. Most of the previously cited works are based on a comprehensive approach integrating various omics. Depending on the needs, different types of individual omics approaches can be integrated with each other. Mainly, genomics, transcriptomics, and proteomics are often used as complementary methods because they connect genetic information (genome), gene expression patterns (transcriptome), and protein abundance and function (proteome), and they provide distinct layers of information about biological systems and a more complete understanding of complex biological processes. The majority of the previously mentioned works include a multi-omics approach.

## 6. Conclusions

Microorganisms play a crucial role in the bioremediation of environments contaminated with various hazardous substances. A large group of such pollutants are substances generated by the textile industry, which, in the face of growing consumerism, driven by the culture of fast fashion, uses millions of tons of chemicals at each stage of textile production. Dyes are a special group of chemical compounds associated with the textile industry. Due to their function (the need to maintain color), they are characterized by high durability and resistance to biological decomposition and physicochemical conditions. Therefore, their removal from contaminated environments poses significant problems. The use of the metabolic activity of microorganisms for the degradation and detoxification of dyes is a promising area for research and implementation of practical solutions. However, in order to be able to implement technology using bacteria and microscopic fungi into the processes of cleaning dye-contaminated environments, it is necessary to have a detailed and in-depth understanding of the mechanisms responsible for this process, to study changes in microbial metabolism under the influence of dyes, and to understand cell physiology and cellular strategies to overcome the stress caused by toxic dyes. Such a thorough analysis of the processes occurring in microbial cells in response to external factors is possible only thanks to the use of advanced omics techniques such as genomics, transcriptomics, proteomics, and metabolomics. Omics methods (especially multi-omics) provide an integrative understanding of the whole biodegradation process, which helps efficient processes for the bioremediation of dye-contaminated areas be designed. This review describes recent advances in the use of microorganisms to remove textile dyes from the environment, mainly focusing on studies using omics technologies. Without detailed information about the genome, transcriptome, proteome, and metabolome of microorganisms, it is impossible to completely decipher the processes occurring in cells and thus develop effective bioremediation strategies using microorganisms. The main problem that may arise during omics research is the interpretation and management of big data. Omics techniques generate huge amounts of data, which can pose challenges in analyzing, interpreting, and processing and require advanced bioinformatics and statistical tools. Also, interdisciplinary knowledge and approaches are essential for understanding the complex interactions between different omics and their connection with biological functions. Another problem that may arise is the standardization of methods and procedures used to ensure the maximum reproduction of omics results. However, further technological development may allow us to overcome emerging problems and shortcomings in omics research. The improved bioinformatics tools for data integration, machine learning algorithms for pattern recognition, and enhanced computational power will facilitate more effective data analysis and interpretation. It is also necessary to expand and refine omics databases, especially for non-model organisms. This will enhance the quality and applicability of omics data. All these technological advancements combined with increased accessibility to omics technologies are expected to reduce the costs of omics-based research. Further combining omics data with synthetic biology approaches may lead to the engineering of microorganisms with enhanced dye-decolorizing capabilities.

The previously mentioned challenges faced by omics-based approaches in dye decolorization research mean that this modern path of understanding the processes occurring in microorganisms in the presence of dyes is not always applicable. However, it is believed that advances in bioinformatics, comprehensive databases, cost reductions, integration with synthetic biology, improved microbial community surveys, and multi-omics approaches have the potential to significantly advance this field and, ultimately, enable more efficient, cost-effective, and holistic bioremediation strategies of textile dyes.

## Figures and Tables

**Table 1:** List of dye-decolorizing bacterial and fungal strains with genome sequenced.

Strain	Database	Accession Number	The Place of Isolation	Decolorization Capability	References
*Kocuria indica* DP-K7	DDBJ/ENA/GenBank	WMHZ00000000	Sediment sample; Chorao Island, India	Decolorization of 68% Methyl Red after 160 h of incubation.	[81]
*Shewanella* sp. LC2	GenBank	VFSJ00000000	Textile industry wastewater effluent; Peru	Decolorization of 90% to 97% Direct Blue 71, Remazol Red, and Remazol Yellow Gold after 24 h of incubation	[82]
*Shewanella* sp. LC6	GenBank	VFSK00000000			
*Pseudomonas aeruginosa* PFK10	GenBank	AZBM00000000	Effluent treatment plant; Gujarat, India	Capable of degrading various mono-azo, di-azo, tri-azo, and poly-azo textile dyes utilized in dyeing and printing industries	[83]
*Shewanella xiamenensis* BC01	GenBank	JGVI00000000	Seawater sample; Xiamen, China	Decolorization of textile azo dyes and production of nanowires in cells, responsive to electron transfer, thereby accelerating azo dye reduction	[84]
*Proteus hauseri* ZMd44	GenBank	AWXP00000000	Lanyang Plain, Taiwan	Utilized for applications in microbe fuel cells with wastewater using azo dye Reactive Blue 160	[85]
*Bacillus amyloliquefaciens* AD20	PATRICbrc	1390.742	Dye waste pond near a textile factory; Nigeria	Exhibited Azo dye decolorization ability	[86]
*Bacillus subtilis* C3	DDBJ/EMBL/GenBank	JYOG00000000	Effluent treatment plant; Gujarat, India	Displayed approximately 95% to 100% decolorization of various azo dyes during 24 to 48 h of incubation under static culture conditions	[87]
*Priestia aryabhattai* BD1	DDBJ/ENA/GenBank	JANIPC000000000	Dye waste sediment behind a textile company; Lagos, Nigeria,	Decolorization of Cibacron dyes—Reactive Blue 4% Red and Brilliant Yellow 6% Green	[88]
*Novibacillus thermophiles* SG-1	GenBank	CP019699.1	Saline soil sample; Guangdong Province, China	Orange I decolorization	[89]
*Anoxybacillus* sp. PDR2	GenBank	CP047158	Soil near a textile dye manufacturing factory; China	Decolorization of 82–98% of Direct Black G (100–600 mg/L) within 48 h	[90]
*Shewanella algae* 2NE11	GenBank	CP055159	Olive processing company effluent; Tacna, Peru	Decolorization of azo and anthraquinone dyes with a decolorization rate of 89–97%	[91]
Consortium of bacteria: *Stenotrophomonas acidaminiphila* APG1, *Pseudomonas stutzeri* APG2, and *Cellulomonas* sp. APG4	GenBank	APG1: JAACYG000000000 APG2: JAACYH000000000 APG4: JAACYI000000000	Sediment of the Alang ship recycling yard, Gujarat, India	Degradation of the mono-azo dye, Reactive Blue 28, and the aromatic amines released upon the cleavage of azo bond	[92]
Consortium of halo-thermophilic bacteria *Alteribacillus* as the dominant genus	nf.	nf.	nf.	Significant decolorization ability under a wide range of salinity (1–10%), pH (7–9), and temperature (45–60 °C)	[93]
*Pantoea ananatis* Sd-1	GenBank	AZTE00000000	Endophytic bacteria from rice seeds; China	Decolorization of various synthetic dyes	[94]
*Tenacibaculum* sp. HMG1	GenBank	LDOD00000000	Deep sea sediment; Pacific Ocean	Almost complete decolorization of Malachite Green within 12 h	[95]
*Brevibacterium limosum* sp. Nov., *Brevibacterium pigmenatum* sp. Nov., *Brevibacterium atlanticum* sp. Nov.	GenBank	*B. limosum*: KU560320 *B. pigmenatum*: KU560298 *B. atlanticum*: MN463008	Sediment samples; South Atlantic and Western Pacific	Decolorization of Congo Red, Toluidine Blue, and Reactive Blue	[96]
*Streptococcus thermophilus* CGMCC 7.179	GenBank	KR106994	Fermented dairy products; Inner Mongolia	Production of peroxidase with the ability to decolorize Reactive Blue 5.	[97]
*Halomonas pacifica* M31 and *Shewanella algae* M69b	GenBank	M31: JAQQYI000000000 M69b: JAQQYH000000000	Uninhabited coastal Red Sea region; Saudi Arabia	A 77% decolorization of dyes in artificial textile effluent derived from a mixture of Indigo carmine, Malachite Green, Cotton Blue, Bromocresol Green, and Reactive Red 66	[98]
*Pseudomonas stutzeri*, AK6	GenBank	PZYR00000000	Textile effluent collected from the dye manufacturing industry; Gujarat, India.	Decolorization of 86% of Acid Blue 113 within 96 h.	[99]
*Salinivibrio kushneri* HTSP	GenBank	PXUD00000000	Salt pan; Marakkanam, India	More than 80% decolorization within 48 h for Coomassie Brilliant Blue G-250 (500–3000 mg/L), Safranin, and Congo Red (50–800 mg/L)	[100]
*Schizopora paradoxa* KUC8140	DDBJ/EMBL/GenBank	LBNM00000000	Oakwood decay; Korea	Remazol Brilliant Blue R decolorization activity	[101]
Schizophyllum commune IUM1114-SS01	GenBank	JAATOI000000000	The Culture Collection of Mushrooms at Incheon National University	Dye decolorization ability from 50% up to 97% with the dyes: Crystal Violet, Amaranth Red, Brilliant Blue G, and Congo Red	[102]

nf.—not found.

**Table 2:** Microbial strains and their proteins were identified via proteomics study and proposed as being involved in dye decolorization.

Microorganism	Dye(s)	Technique Used	Identified Proteins Involved in Decolorization	References
*Anoxybacillus* sp.	Direct Black G	Tandem Mass Tag quantitative proteomics technology	Thioredoxin reductase	[115]
*Proteus hauseri* ZMd44	Reactive Blue 160	nano-LC-MS/MS	Laccase, dehydrogenase, and porin	[116]
*Shewanella seohaensis* NIODMS14	Reactive Black 5, Reactive Green 19 Reactive Red 120	LC-MS-QTOF	Oxidoreductases and an alkyl hydroperoxide reductase	[111]
*Leptosphaerulina* sp. CECT (20913)	Reactive Black 5	Nano-LC-ion trap mass spectrometry	Oxidoreductases, peroxidases, flavo-oxidases, and laccases	[110]
*Irpex lacteus* CD2	Direct Red 5	Multidimensional Protein Identification Technology	Peroxidases	[105]
*Lentinus squarrosulus* AF5	Dye mix consisting of: Acid Black 10B, Reactive Black 5, Reactive Blue 160	LC-QTOF-MS	Peroxidase and oxidoreductase	[112]
*Myrothecium roridum* IM6482	Acid Blue 113, Acid Red 27, Direct Blue 14, Acid Orange 7	2D-LC-MS/MS	Laccase	[113]
A thermophilic microflora	Direct Black G	2D-LC-MS/MS	NADH-ubiquinone reductase and NADH-quinone oxidoreductase	[117]
*Pseudomonas putida* A514	na.	LC-MS -	Peroxidases	[118]
*I. lacteus* SSF and *I. lacteus* SmF	Azo and anthraquinone dyes	MALDI-TOF-MS/ MS	Dye-decolorizing and manganese-oxidizing peroxidases	[119]
*S. decolorationis* S12	Amaranth	2-DE and MALDI-TOF/TOF/MS	nucleoside diphosphate kinase, ATP-dependent Clp protease proteolytic subunit, and N-acetyl-gamma-glutamylphosphate reductase	[114]

na.—not applicable.

## Data Availability

Not applicable.
